# Methodology for Assessing the Level of Biological Risk of the Emergence and Spread of Infectious and Parasitic Potato Diseases Based on Environmental Stability Parameters

**DOI:** 10.3390/biology15211977

**Published:** 2026-11-02

**Authors:** Alexey Dorokhov, Nikolay Sazonov, Yulia Lyashchuk, Vladimir Teterin, Nikolay Panferov, Alexey Ovchinnikov

**Affiliations:** Department of Machine Technologies for Cultivation and Harvesting of Open-Ground Vegetable Crops, Federal Scientific Agroengineering Center VIM, 109428 Moscow, Russia; dorokhov.vim@yandex.ru (A.D.); beautifulstranger1306@gmail.com (Y.L.); v.s.teterin@mail.ru (V.T.); nikolaj-panfyorov@yandex.ru (N.P.); aleksovchinn@gmail.com (A.O.)

**Keywords:** environmental stability, infectivity, pathogens, persistence, soil, survivability, virulence, water

## Abstract

This study is devoted to the quantitative assessment of the level of biological risk of the emergence and spread of infectious and parasitic potato diseases based on environmental stability parameters. The study examined the survival conditions of pathogens in soil, water systems, and on various environmental objects, classified according to the criterion of internal and external localization of pathogens. In this study, we conducted a scoring and rating analysis of the threats of potato infection by pathogens of bacterial, viral, helminth, and mycotic etiology, which pose a danger in terms of infection and death of plantings, significant yield losses, as well as deterioration of the quality of commercial and seed potato tubers. Particularly dangerous are species characterized by high survival in the external environment while retaining virulent properties. The developed system implied regular monitoring and updating of data on the emergence of pathogens with new forms of resistance. Phytosanitary control in the context of global trade requires systematization of available data on pathogens of infectious and parasitic potato diseases in order to promptly identify pathogens and prevent their further spread. The use of a scoring and rating approach increases the objectivity of phytosanitary risk assessment and can serve as a scientific basis for managerial decision-making aimed at reducing the epiphytotic potential of pathogens and ensuring stable potato production.

## Simple Summary

Potato crops can be attacked by many different disease-causing organisms, including bacteria, viruses, fungi, and tiny worms. These diseases reduce harvests and worsen tuber quality, and some can spread quickly through soil, water, farm equipment, or seed potatoes. Farmers and regulators need a practical way to judge which threats are most serious so they can act early. This study developed a scoring method that rates how well each disease-causing organism can survive outside the potato plant—in water, in soil, and on objects such as tools, storage floors, and containers. We applied this method to 46 potato pathogens. The results showed that fungi and certain soil-dwelling bacteria pose the greatest risk because they can survive for a long time and remain capable of causing infection. Soil and contaminated equipment were more important than water for spreading these diseases. The risk scores also matched independent field data on how often tubers were actually infected. This simple scoring system can help authorities and growers prioritize monitoring and control measures, reduce disease spread, and support more stable potato production.

## 1. Introduction

The environmental persistence of pathogens is directly linked to the mechanisms and routes of their transmission to plants and to their resistance to the plant’s immune response. This study is the third work in the series “Rating analysis of the level of biological risks of the emergence and spread of infectious and parasitic potato diseases.” The first work in the series was devoted to the quantitative assessment of the level of biological risk of the emergence and spread of infectious and parasitic potato diseases based on the parameters of transmission mechanisms and pathways of their pathogens [1]. The second study examined approaches to the quantitative assessment of the level of biological risk of the emergence and spread of infectious and parasitic potato diseases (IPPD) based on the parameters of pathogenicity and resistance in the host organism [2]. The present work continues the topic of scoring and rating analysis of threats of potato infection by pathogens of bacterial, viral, helminth, and mycotic etiology from the perspective of their environmental stability. The environmental stability of pathogens of infectious and parasitic potato diseases reflects their ability to survive outside the plant organism due to various mechanisms of adaptation to changing environmental conditions.

This study examines the environmental persistence of pathogens causing potato diseases, including in aquatic systems, soil, and various environmental objects (plant debris, tubers, manure, personnel clothing and personal protective equipment, agricultural implements and machinery, harvesting equipment, potato storage flooring, and containers and packaging materials) [1,2].

*Dickeya solani* and *Dickeya dianthicola* are characterized by low pathogenicity for three insect species (*Drosophila melanogaster*, *Sitophilus oryzae*, and *Spodoptera littoralis*) and can persist for some time in their living and dead bodies, posing a threat in terms of external introduction of diseases into previously unaffected territories [3,4,5,6,7,8,9,10].

Free-living inhabitants of water systems pose a threat to potato crops during irrigation, stormwater runoff during heavy rains, and flooding of fields during spring floods. At the same time, for some pathogens, total moisture (soil moisture and humidity combined) acts as a factor of spread; for example, amoeboids and plasmodia (several “fused” amoeboids) of the myxomycete (plasmodiophoromycete) *Spongospora subterranea* can move only under conditions of high soil moisture, including transportation with rainwater flows [11,12].

It should also be noted that for a number of pathogens of fungal etiology, total moisture is an essential factor for their growth, including: *Helminthosporium solani* [13,14], *Synchytrium endobioticum* [15,16], *Rhizoctonia solani* [17], *Endomyces geotrichum* (*Geotrichum candidum*) [18,19], *Phytophthora infestans* [20,21], *Phoma exigua* (*Ophiobolus porphyrogonus*) [22,23], *Fusarium* spp. (*F. sambucinum*, *F. solani*, *F. avenaceum*, *F. oxysporum*) [24,25], *Pythium ultimum* [26,27], *Ascochyta lycopersici Brunaud* (*Didymella lycopersici*) [28], *Sclerotinia sclerotiorum* [29], *Erysiphe cichoracearum* [30], *Botrytis cinerea* (*Botryotinia fuckeliana*) [31,32], and *Passalora concors* [33].

*Pseudomonas aeruginosa* is a bacterium with high adaptive plasticity to various environmental conditions. Bacteria of the genus *Pseudomonas* freely inhabit water systems and soil [34,35].

For the bacteria *Clostridium perfringens* [36] and *Bacillus cereus* [37], soil is the primary habitat. The spores of these bacteria can persist in soil for years: spores of *Clostridium perfringens* persist for more than 10 years, while spores of *Bacillus cereus* can persist for up to 30 years, maintaining virulence, the potential to germinate into vegetative (parental) forms, and the ability to colonize the host organism.

The potato rot nematode *Ditylenchus destructor Thorne* can persist in the form of an adult for several years by entering a state of anabiosis, while its eggs overwinter in the soil and become active under favorable conditions [38,39].

Cysts of *Globodera rostochiensis* [40,41] and *Globodera pallida* [40,41,42] can be transported by water flows without harm to the larvae, while in soil the larvae within cysts remain viable for up to 8–9 years. However, in the absence of a suitable host plant nearby, only a small proportion of larvae emerge from the cysts and subsequently die, while the rest remain in a state of anabiosis until more favorable conditions arise. Eggs and larvae of *Meloidogyne hapla Chitwood* [43] can overwinter in soil and in potato neoplasms together with the tuber.

Most viruses infecting potatoes are not typical inhabitants of soil and water systems; however, they are able to persist in living plant tissues, including planting material [44]. The only exception is *Potato mop-top virus* (*PMTV*), which is a symbiotic pathogen associated with the reproductive system of *Spongospora subterranea*. The possibility of such a symbiotic association is due to the unique characteristics of these pathogens.

*Potato mop-top virus* is a phytopathogen that causes disturbances in potato organogenesis (mop-top symptoms of leaves, shortening of internodes, and local necrotic processes caused by metabolic disruption). At the same time, the virus is completely harmless to *Spongospora subterranea* and is capable of symbiosis. *Spongospora subterranea* is a plasmodiophoromycete (combining morphophysiological features of fungi and bacteria) that causes powdery scab of potato. Due to this unique and rare combination, *Potato mop-top virus* and *Spongospora subterranea* form a unified system for the spread of infection. Spores of *Spongospora subterranea* carrying PMTV can persist in soil for up to 18 years, retaining virulent *Potato mop-top virus* [44,45].

The aim of the study is to conduct a quantitative assessment of the biological risk of potato infection by pathogens of infectious and parasitic diseases based on their resilience in the external environment. This includes their ability to survive outside the plant host, mechanisms of adaptation to changing conditions (total moisture, temperature, living vectors, soil, and water systems), as well as the duration of viability and virulence in dormant forms (spores, eggs, cysts).

Despite this body of literature [3,4,5,6,7,8,9,10,11,12,13,14,15,16,17,18,19,20,21,22,23,24,25,26,27,28,29,30,31,32,33,34,35,36,37,38,39,40,41,42,43,44,45], no previous study has combined these qualitative resilience descriptors into a quantitative, verifiable scoring system, nor tested whether such scores are consistent with independently collected field surveillance data. This gap motivates the present work. We hypothesize that a composite resilience score, derived systematically from literature-reported survival and virulence-retention parameters, will correlate significantly with independently recorded field infection levels, thereby providing a verifiable, rather than purely expert-based, basis for phytosanitary risk classification.

## 2. Materials and Methods

The resilience of pathogens of infectious and parasitic potato diseases in the external environment reflects their ability to survive outside the host organism through a variety of adaptive mechanisms to changing living conditions.

Analysis of studies by Russian and foreign researchers on the mechanisms of pathogen resilience [46,47,48,49,50,51,52,53,54,55,56,57,58,59,60,61,62], as well as our own previous research [63,64,65,66,67,68,69], allowed us to identify key patterns linking species-specific characteristics of pathogens with their level of environmental resilience.

The ability to survive, reproduce, and maintain infectivity (virulence) in a given habitat depends on the individual traits of the pathogens, their structure, physiology, and life cycle. The formation of biofilms and capsules facilitates the adaptation and survival of mature pathogen forms, while the production of cysts, spores, and eggs ensures the continuation of the life cycle and the generation of new individuals even after the death of the parent form.

For a comprehensive analysis, the study considered survival conditions in soil, water systems, and various environmental objects, classified according to two criteria: the internal presence of pathogens (in water, soil, plant residues, tubers, manure, and other organic and inorganic objects) and the external presence of pathogens (on agricultural tools, storage facility floors, burlap, crate surfaces, and other objects, including personal protective equipment, clothing, and footwear of personnel).

From the perspective of infecting healthy plants, the most dangerous are free-living soil inhabitants, which can cause disease in freshly planted crops and significantly impact future yields due to plant death and necrotic decay of tuber tissues. This group of pathogens can only be controlled through regular soil monitoring and testing for phytopathogens and parasites in cases of severe crop losses.

Free-living inhabitants of water systems pose a risk during irrigation, runoff from heavy rains, or flooding of fields during spring inundations.

Pathogens that persist on agricultural machinery, transport vehicles, as well as within seed material, plant residues, manure, mineral fertilizers, and other organic and inorganic objects that can facilitate pathogen entry into soil, pose a risk in terms of external introduction of diseases to previously healthy areas.

### 2.1. Data Collection

To assess the dependence of the spread of risk factors on the level of biological risk in terms of resilience parameters in the external environment, statistical data from the Rosselkhoztsentr on the volume of spread of seed tubers of pathogens characteristic of Russia in the period from 2020 to 2024 were analyzed in 76 constituent entities of the Russian Federation (Moscow Region, Kursk Region, Oryol Region, Ryazan Region, Voronezh Region, Kaluga Region, Belgorod Region, Smolensk Region, Lipetsk Region, Tver Region, Tula Region, Kostroma Region, Bryansk Region, Vladimir Region, Ivanovo Region, Yaroslavl Region, Kaliningrad Region, Arkhangelsk Region, Pskov Region, Vologda Region, Leningrad Region, Novgorod Region, Republic of Karelia, Republic of Komi, Republic of Adygea, Volgograd Region, Krasnodar Region, Astrakhan Region, Rostov Region, Republic of Kalmykia, Stavropol Territory, Chechen Republic, Republic of Dagestan, Republic of Karachay-Cherkessia, Republic of North Ossetia-Alania, Republic of Ingushetia, Republic of Kabardino-Balkaria, Samara Region, Republic of Tatarstan, Ulyanovsk Region, Penza Region, Republic of Mari-El, Chuvash Republic, Republic of Mordovia, Republic of Bashkortostan, Kirov Region, Perm Region, Nizhny Novgorod Region, Republic of Udmurtia, Saratov Region, Orenburg Region, Kurgan Region, Tyumen Region, Chelyabinsk Region, Sverdlovsk Region, Kemerovo Region, Irkutsk Region, Republic of Tuva, Altai Territory, Omsk Region, Novosibirsk Region, Republic of Khakassia, Krasnoyarsk Territory, Altai Republic, Tomsk Region, Amur Region, Primorsky Territory, Zabaykalsky Krai, Republic of Sakha (Yakutia), Republic of Buryatia, Khabarovsk Territory, Jewish Autonomous Okrug, Magadan Region, Murmansk Region, Tambov Region, and Sakhalin Region) [70,71,72,73,74,75].

### 2.2. Statistical Analysis 

A brief characterization of biological risk factors in terms of resilience in the external environment is presented in Table 1.

The authors used a comprehensive approach to conduct the study, including a literature review, statistical analysis, and expert assessments. Databases were created in Microsoft Excel to process the results.

As shown by the analysis of literature sources [3,4,5,6,7,8,9,10,11,12,13,14,15,16,17,18,19,20,21,22,23,24,25,26,27,28,29,30,31,32,33,34,35,36,37,38,39,40,41,42,43,44,45,46,47,48,49,50,51,52,53,54,55,56,57,58,59,60,61,62] and the results of our own studies [63,64,65,66,67,68,69], infectious and parasitic potato diseases are characterized by the following resilience parameters:

- Favorability of the considered habitat for pathogen activity;
- Survival in the considered habitat;
- Duration of virulence preservation in the considered habitat.

During the analysis, parameters for assessing the resilience factors of biological risk in the external environment were identified (Table 2).

Scoring of resilience parameters was performed by a panel of five experts from the author team based on a systematic review of the literature sources cited above and the authors’ own field observations accumulated in prior studies of the series. Discrepancies in individual assessments were resolved through iterative discussion among the authors until consensus was reached for each pathogen.

The assessment of the biological risk level based on resilience parameters in the external environment was carried out using the following formula:

*L_env_* = *L_w_* + *L_s_* + *L_obj_* (1)

Here, *Lenv* is the level of pathogen resilience risk in the external environment, *Lw* is the level of pathogen resilience in water, *Ls* is the level of pathogen resilience in soil, *Lobj* is the level of pathogen resilience among other environmental objects.

The level of biological risk based on resilience parameters in the external environment (Lenv) is characterized according to the following scale:

<2 points—very low risk (VLR);
2 to <4—low risk (LR);
4 to <7—medium risk (MR);
7 to <10—high risk (HR);
≥10—very high risk (VHR).

In order to identify the influence of resilience in water, soil, and other environmental objects on the overall group risk level, their weighting coefficients were calculated using the following formulas:

λ*_w_* = *L_w_*/*L_env_* (2)

λ*_s_* = *L_s_*/*L_env_* (3)

λ*_obj_* = *L_obj_*/*L_env_* (4)

Here, *λw*, *λs*, and *λobj* are the weighting coefficients for the levels of resilience in water, soil, and other environmental objects, respectively.

Calculations were performed in STATISTICA (StatSoft Inc., Tulsa, OK, USA, version 12). Statistical analysis was performed using Pearson’s linear correlation coefficient (r) to assess the relationship between biological risk scores and empirical pathogen prevalence data. The significance of correlation coefficients was evaluated using Student’s *t*-test:

(5) $$t=r\sqrt{\frac{n-2}{1-r^{2}}}$$

Here, r is the sample Pearson correlation coefficient and n is the sample size. Statistical significance was set at *p* < 0.05.

To assess the robustness of the proposed risk classification scale, a sensitivity analysis was performed by shifting the category thresholds by ±1 point and re-classifying all 46 analyzed pathogens. The proportion of pathogens retaining their original risk category was used as an indicator of classification stability.

To further evaluate whether transmission-pathway risk provides independent explanatory power beyond environmental resilience, a multiple linear regression model was additionally fitted, with the average annual proportion of infected tubers as the dependent variable. Multicollinearity between predictors was assessed using the variance inflation factor.

## 3. Results

### 3.1. Distribution of Pathogens Across Environmental Resilience Risk Categories

Based on the analysis of resilience parameters of pathogens causing infectious and parasitic potato diseases in the external environment, a quantitative assessment of the biological risk level was obtained, the results of which are presented in Table 3.

**Figure 1:**
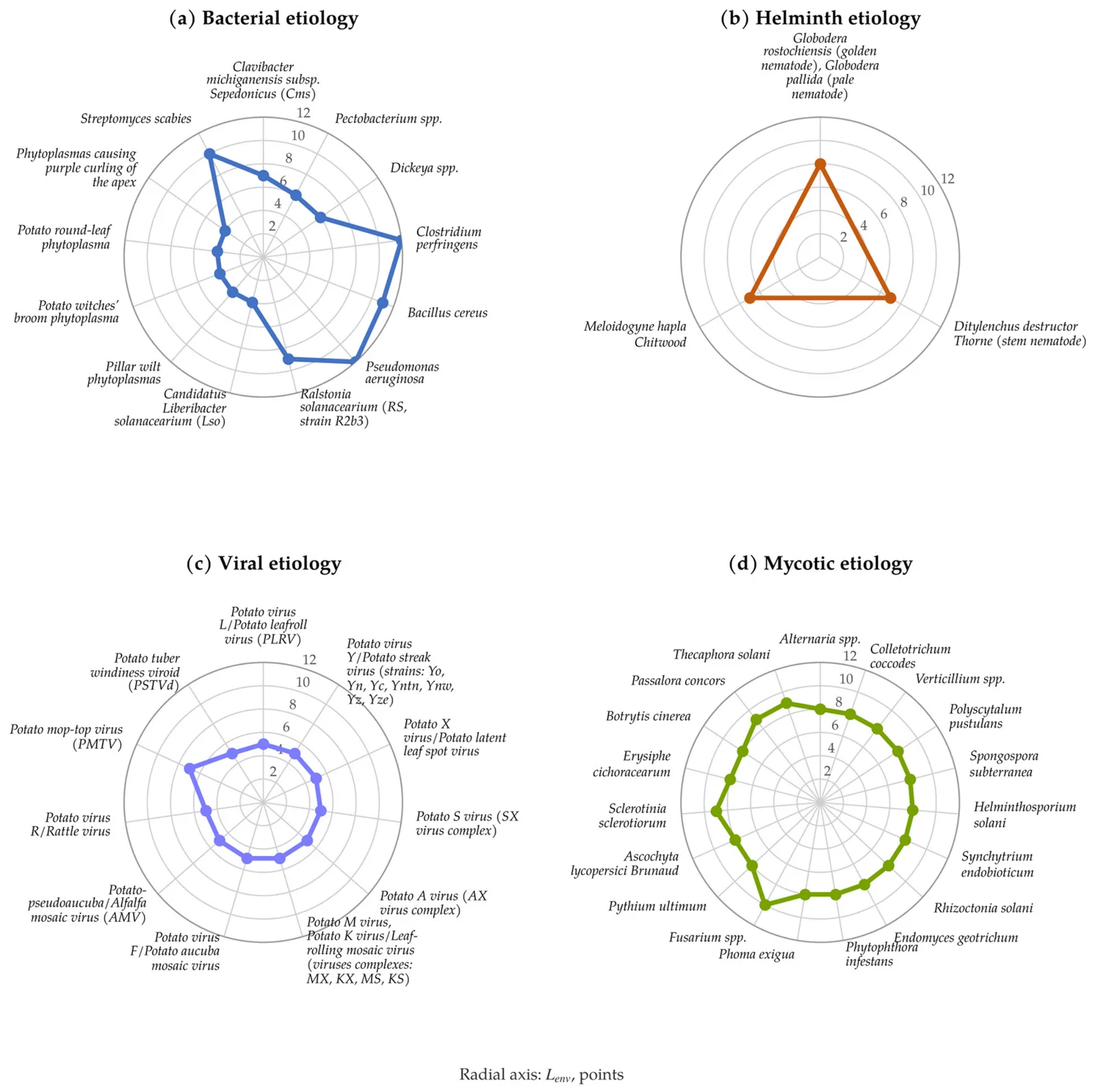
Distribution of the biological risk level based on resilience parameters in the external environment for pathogens of (**a**) bacterial, (**b**) helminth, (**c**) viral and (**d**) mycotic etiology.

Based on the obtained data, graphical distributions of the biological risk level according to resilience parameters in the external environment for pathogens of different etiologies were constructed, as presented in Figure 1.

The distribution of the biological risk level based on resilience parameters in the external environment for pathogens of bacterial etiology is shown in Figure 1a.

The analysis of the data shows that for the largest share of bacterial IPPD pathogens (38.4%), a low level of risk based on resilience parameters in the external environment is characteristic. For 23.1% of pathogens, a medium level of risk is typical, while 15.4% are characterized by a high level of risk. A very high level of risk based on resilience parameters in the external environment is observed in 23.1% of IPPD pathogens of bacterial etiology.

The average score for the group is 7.2. For the pathogens of IPPD of bacterial etiology, the weighting coefficients of resilience in water (*λ_w_* = 0.266), in soil (*λ_s_* = 0.341) and environmental objects (*λ_obj_* = 0.392) fall within a relatively similar range.

The distribution of the biological risk level based on resilience parameters in the external environment for pathogens of helminth etiology is presented in Figure 1b.

The analysis shows that for 66.7% of pathogens of IPPD of helminth etiology, a medium level of risk bordering on high is characteristic (the average group score is 7.3), while the remaining 33.3% are characterized by a high level of risk based on resilience parameters in the external environment.

For the pathogens of IPPD of helminth etiology, the weighting coefficients of resilience in water (*λ_w_* = 0.226), in soil (*λ_s_* = 0.363) and environmental objects (*λ_obj_* = 0.411) increase according to resilience characteristics, reflecting the importance of soil and various environmental objects, including agricultural equipment, containers, and storage facility floors, in pathogen persistence.

The distribution of the biological risk level based on resilience parameters in the external environment for pathogens of viral etiology is presented in Figure 1c.

The analysis shows that pathogens of IPPD of viral etiology are characterized by a medium level of risk based on resilience parameters in the external environment. The average score for the group is 5.2, corresponding to a medium risk level. For the pathogens of IPPD of viral etiology, the weighting coefficients of resilience in water (*λ_w_* = 0.195), in soil (*λ_s_* = 0.221) and environmental objects (*λ_obj_* = 0.584) reflect the high importance of environmental objects in pathogen persistence.

The distribution of the biological risk level based on resilience parameters in the external environment for pathogens of mycotic etiology is presented in Figure 1d.

The analysis shows that pathogens of IPPD of mycotic etiology are characterized by a high level of risk based on resilience parameters in the external environment. The average score for the group is 8.3, corresponding to a high-risk level.

The weighting coefficients of resilience in water (*λ_w_* = 0.237), in soil (*λ_s_* = 0.393) and environmental objects (*λ_obj_* = 0.370) reflect the high importance of soil in the persistence of mycotic IPPD pathogens.

**Figure 2:**
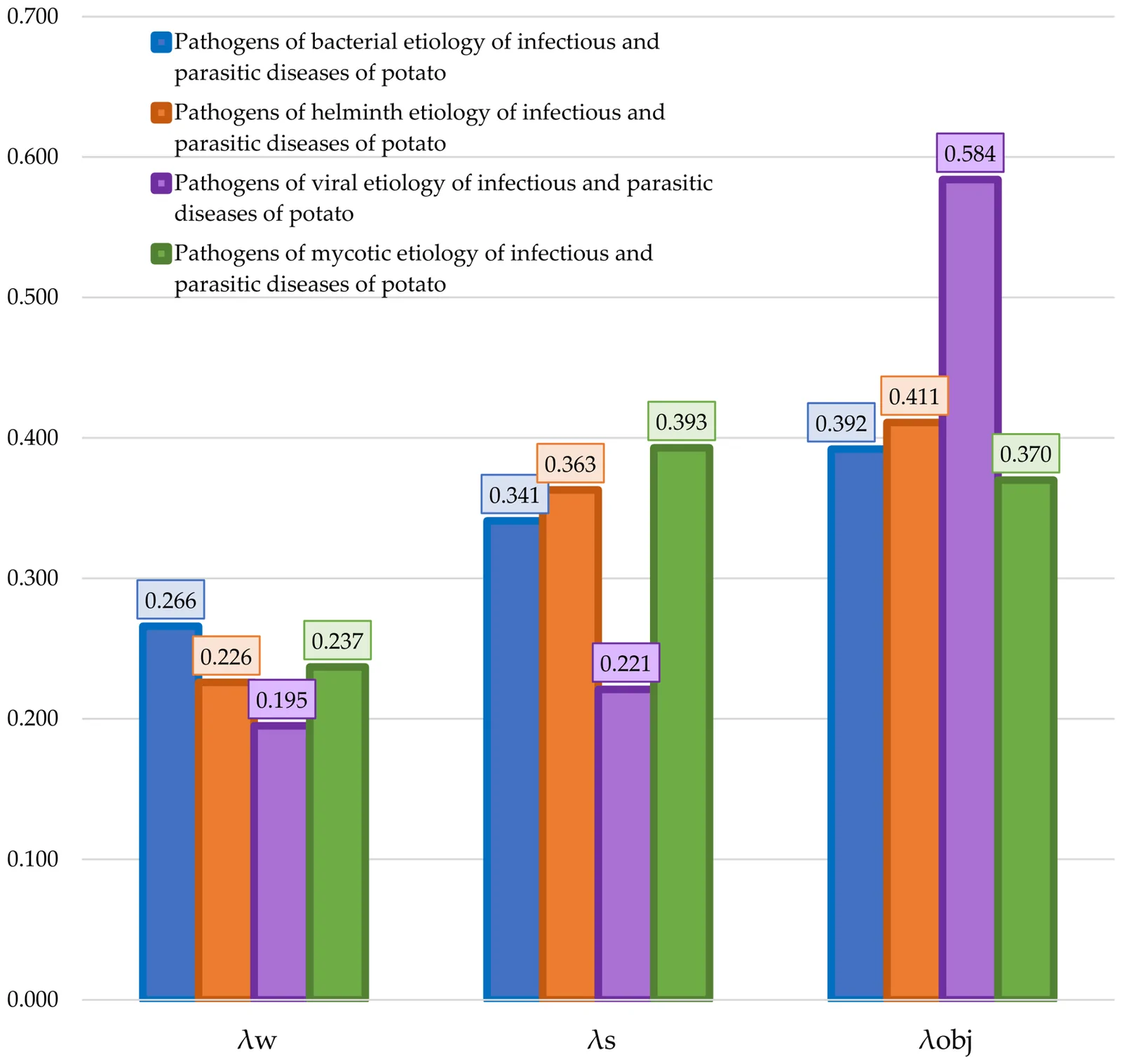
Ratio of weighting coefficients of resilience in the external environment for IPPD pathogens.

The ratio of weighting coefficients of resilience in the external environment for IPPD pathogens is presented in Figure 2.

As shown in Figure 2, soil and environmental objects are of greater importance for the persistence of IPPD pathogens than water systems. Special attention should be paid to viruses and viral complexes, which can persist for long periods in planting material, including in a latent form.

The quantitative assessment of the biological risk level based on resilience parameters in the external environment is presented in Table 4.

The analysis of the data shows that a low level of risk based on resilience parameters in the external environment is characteristic of 10.9% of the considered pathogens of infectious and parasitic diseases of potato, a medium level of risk is characteristic of 34.8%, a high level of risk of 47.8%, and a very high level of risk of 6.5% of the considered pathogens of IPPD.

### 3.2. Sensitivity Analysis of the Risk Classification Scale

Sensitivity analysis showed that a downward shift of the category thresholds preserved the original classification for 76.1% of pathogens (35 of 46), whereas an upward shift preserved classification for 41.3% of pathogens (19 of 46); 17.4% of pathogens (8 of 46) remained stable under both directions. The reduced stability under the upward shift reflects a cluster of pathogens with resilience scores located precisely at the original category boundaries (*Lenv* = 5 and *Lenv* = 8) rather than a systematic weakness of the scoring approach, indicating that classification instability is concentrated among borderline cases. Re-classification was performed on the pathogen-level data in Table 3.

The analysis of the data in Table 4 shows that there are certain patterns between resilience parameters in the external environment and the taxonomic affiliation of pathogens.

In the group with a low level of risk based on resilience parameters in the external environment, all pathogens of infectious and parasitic potato diseases have a bacterial etiology. Pathogens of viral, helminth, and mycotic etiology are absent in this group. The average score for the low-risk group is 4.

In the group with a medium level of risk based on resilience parameters in the external environment, the majority of pathogens of IPPD (68.75%) have a viral etiology, 18.75% have a bacterial etiology, and 12.50% have a helminth etiology. Pathogens of mycotic etiology are absent in this group. The average score for the medium-risk group is 5.63. It should be noted that this group included all analyzed representatives of viral etiology, which is due to the peculiarities of the vital activity of viruses and viral complexes.

In the group with a high level of risk based on resilience parameters in the external environment, the majority of pathogens of IPPD (86.36%) have a mycotic etiology, pathogens of bacterial etiology make up 9.09% and pathogens of helminth etiology make up 4.55%. Viral pathogens are absent in this group. The average score for the high-risk group is 8.36. This etiological distribution is associated with the high resilience of mycotic pathogens in the external environment.

In the group with a very high level of risk based on resilience parameters in the external environment, all pathogens of IPPD have a bacterial etiology. The average score for the very high-risk group is 11.67. This group is represented by particularly dangerous pathogens that are free-living inhabitants of the soil.

The analysis of the proportional etiological structure of pathogens of IPPD showed that mycotic pathogens pose the greatest danger in terms of resilience in the external environment. They account for 41.30% of all analyzed pathogens and form the largest share of the high-risk group.

### 3.3. Correlation, Regression, and Independent Field-Level Validation

It should be noted that there is a correlation between the risk level of infection with infectious and parasitic potato diseases based on transmission mechanisms and pathways (*Ltr*) and resilience in the external environment (*Lenv*), as presented in Table 5.

The analysis of the risk level of infection based on transmission mechanisms and pathways (*Ltr*) was presented by the authors in the first study of the series “Rating analysis of the level of biological risks of the emergence and spread of infectious and parasitic potato diseases” [1].

A very strong negative linear correlation was found between the risk level based on transmission mechanisms and pathways and the risk level based on environmental resilience, with r = −0.890, *p* = 0.0003. This finding is consistent with a compensatory survival strategy, whereby a reduced number of active transmission pathways is associated with increased environmental resilience. Given the limited sample size, this pattern warrants further verification on an expanded pathogen dataset in subsequent stages of the study.

The dynamics of infection of seed potato tubers by the main pathogens of infectious and parasitic diseases are presented in Table 6.

**Figure 3:**
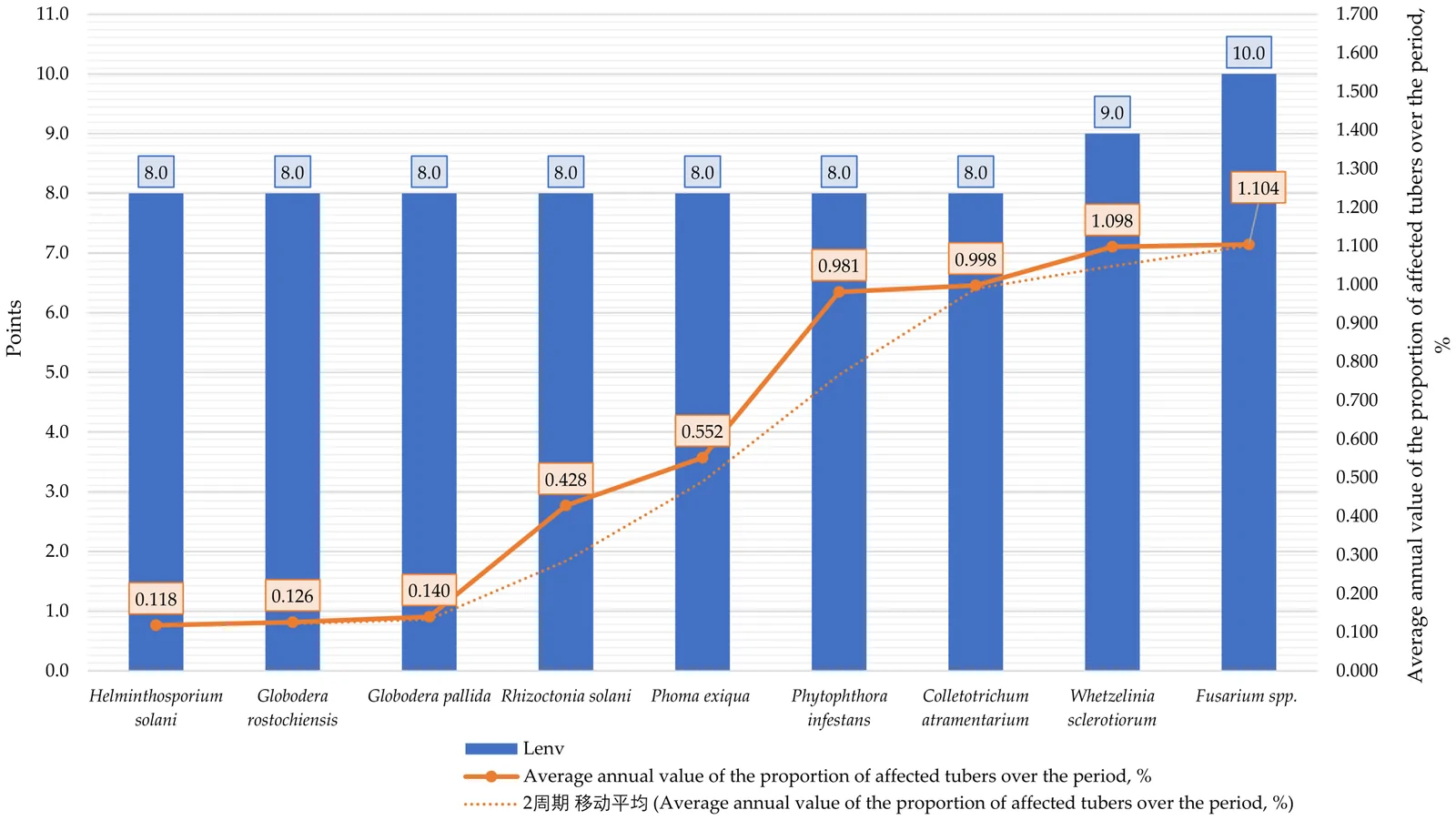
Correlation between the level of biological risk for the parameters of pathogenicity and resistance in the host organism and the average annual proportion of tubers affected by the main pathogens of infectious and parasitic diseases of potatoes.

Using the presented statistical data and point values characterizing the level of biological risk according to the parameters of pathogenicity and resistance in the host organism, a diagram of the relationship between the level of biological risk and the proportion of tubers affected by the main pathogens of IPPD was constructed, presented in Figure 3.

The analysis of the data shows that among the pathogens of infectious and parasitic potato diseases registered in the Russian Federation over the past five years, the total proportion of infected seed tubers (PIT) was 6.647%, of which mycotic pathogens accounted for 6.381% and helminth pathogens for 0.266%.

The leading place in terms of the level of damage caused to seed potatoes is occupied by pathogens of *Fusarium* (dry rot of tubers)—fungi of the genus *Fusarium*: *F. sambucinum*, *F. solani*, *F. avenaceum*, *F. oxysporum* (PIT = 1.104%, L_env_ = 10).

Total moisture is a key factor in the growth and transmission of these pathogens. Sharp fluctuations in temperature and humidity during storage are especially dangerous, as they lead to condensation on tubers. Fungi of the genus *Fusarium* are typical soil inhabitants capable of saprophytic growth on organic residues. Their spores overwinter in soil, infected tubers, and plant residues. These pathogens can persist on agricultural equipment, containers, transport, and in storage facilities if they are not properly cleaned and disinfected after use.

A high positive linear correlation was found between the biological risk level based on environmental resilience parameters and the average annual proportion of infected tubers for 2020–2024, with r = 0.765 across the 10 analyzed pathogens and *p* = 0.0038, confirming that higher environmental-resilience risk scores correspond to greater actual infection levels of planting material.

A multiple linear regression model was additionally fitted to evaluate whether transmission-pathway risk provides independent explanatory power beyond environmental resilience in predicting the average annual proportion of infected tubers. The model explained a moderate proportion of variance, with R^2^ = 0.612, F = 6.30, *p* = 0.023. However, only the environmental resilience score approached significance as an independent predictor, *p* = 0.084, while transmission-pathway risk did not, *p* = 0.693. This finding is consistent with the strong negative correlation between the two variables reported above and was further confirmed by a variance inflation factor of 4.80, indicating substantial multicollinearity between the predictors. It should be noted that these results suggest environmental resilience alone captures most of the predictive information relevant to field infection outcomes, supporting the univariate correlation model as the more parsimonious and interpretable choice for this dataset. The regression was fitted on the paired *Ltr*, *Lenv*, and PIT values listed in Table 5 and Table 6.

As a complementary check, field-level spread data from the same Rosselkhoztsentr monitoring reports were examined for five pathogens or pathogen groups with complete five-year records, including two foliar diseases not covered by the tuber-storage analysis, late blight and early blight, as well as an aggregate viral disease category are presented in Table 7 [70,71,72,73,74].

The direction of association was consistent with the tuber-based analysis, with higher-Lenv pathogens showing greater field-level spread area (r = 0.827, *p* = 0.084), approaching but not reaching conventional statistical significance given the small number of pathogens with directly comparable field-level records.

## 4. Discussion

### 4.1. Comparison with Prior Literature and International Risk-Assessment Frameworks

The analysis of literature sources and research results conducted by us demonstrates the diversity of approaches and the depth of study of issues related to the resilience of potato disease pathogens in the external environment.

The proposed methodology for assessing the level of biological risk of the emergence and spread of infectious and parasitic potato diseases (IPPD) based on resilience parameters in the external environment takes into account the characteristics of 46 pathogens of bacterial, viral, helminth, and mycotic etiology.

The features of resilience of bacterial pathogens in the external environment were examined in the works of Matilla et al. [3], Pédron et al. [4], Ge et al. [5], Ma et al. [6], Crone et al. [34], and Urgancı et al. [35]. Notably, while Matilla et al. and Pédron et al. emphasized the epidemiological role of *Dickeya* spp. primarily through insect-mediated dispersal, the present analysis indicates that their environmental resilience score remains comparatively moderate (MR), suggesting that transmission-pathway diversity, rather than environmental persistence, is the dominant risk driver for this genus—a distinction with direct implications for the choice of preventive measures.

The resilience of helminth pathogens in the external environment was studied in the works of Ni et al. [38], Haraguchi et al. [39], Price et al. [40], Esquibet et al. [41], Wainer et al. [42], and Evlice et al. [43].

Notably, while these authors emphasize long-term cyst and egg dormancy as the primary survival strategy of potato cyst and root-knot nematodes, the comparatively moderate resilience score obtained in the present rating (MR, average 7.3) indicates that this dormancy capacity is concentrated specifically in soil, consistent with the elevated soil-resilience weighting coefficient reported in Section 3.1 (*λs* = 0.363), rather than reflecting broad persistence across water and other environmental objects.

The features of resilience of viral pathogens in the external environment were considered in the works of Ingram et al. [44], Della Bartola et al. [50].

Notably, unlike the bacterial, helminth, and mycotic groups, viral pathogens are entirely dependent on living plant tissue for persistence rather than free survival in soil or water. This dependence is directly reflected in the present results: viral pathogens occupy a narrow, medium-risk score range (MR, average 5.2) and show a disproportionately high weighting coefficient for persistence in environmental objects such as planting material (λobj = 0.584) relative to water or soil, underscoring that seed-tuber sanitation, rather than soil or water management, is the primary control point for this group.

The resilience of mycotic pathogens in the external environment was examined in the works of Kmoch et al. [14], Strydom et al. [11], and Tsror et al. [45].

Notably, the consistently elevated resilience scores observed across nearly all mycotic pathogens examined here (HR, average 8.3, the highest of the four etiological groups) corroborate reports by these authors that spore and sclerotia formation confer exceptional longevity in soil and on plant residues. Combined with the elevated soil-resilience weighting coefficient for this group (*λs* = 0.393, Section 3.1), this positions fungal pathogens as the etiological group of greatest concern for long-term field contamination, ahead of bacteria, helminths, and viruses.

It is worth noting that the proposed scoring approach is conceptually close to internationally recognized pest risk assessment frameworks, in particular the EPPO Prioritization Process and the USDA-APHIS Weed/Pest Risk Assessment, both of which also combine several weighted criteria into a single composite score. However, these frameworks are mainly aimed at evaluating the probability of introduction and establishment of a pest at the country or regional level, whereas the methodology proposed here treats environmental resilience as a separate, standalone risk dimension. This makes it possible to distinguish more precisely between pathogens that share similar transmission pathways but differ considerably in their ability to persist once outside the host organism.

It should be noted that although the methodology was developed and validated using data specific to potato production in the Russian Federation, the underlying scoring framework is not inherently crop-specific, since it is based on the duration of survival and retention of virulence in water, soil, and other environmental objects. In further studies, this approach could be adapted to other vegetatively propagated crops facing similar soil- and water-borne disease pressures, such as sugar beet or vegetable brassicas, provided that the resilience parameters specific to the pathogens of the target crop are re-evaluated accordingly.

### 4.2. Methodological Validity and Consistency with the Study Series

It should be noted that the practical integration of the methodology for identifying the biological risk of the emergence and spread of infectious and parasitic potato diseases involves a systematic assessment of pathogens not only in terms of environmental persistence, which is the subject of this work, but also an assessment of pathogens by the parameters of the nature of the course of the disease depending on the bioecological conditions of potato cultivation, which is the subject of further research within the study series “Rating Analysis of the Level of Biological Risks of the Emergence and Spread of Infectious and Parasitic Potato Diseases.”

The validity of the proposed scoring approach is further supported by its significant correlation with independent field infection data, r = 0.765, *p* = 0.0038 as shown in Section 3, indicating that the resilience-based scale meaningfully reflects actual epidemiological outcomes rather than representing an arbitrary expert construct.

### 4.3. Practical Implications: Regional Variation, Control Costs, and Climatic Sensitivity

It is worth noting that the proposed risk categories were derived using data specific to the Russian Federation and may benefit from regional recalibration when applied to potato-growing areas with different climatic conditions or pathogen strain compositions.

Substantial regional variation further illustrates the practical relevance of the proposed risk categories: while the national average proportion of infected tubers was 3.39% in 2020, individual regions such as the Republic of Khakassia showed proportions as high as 86.83%, indicating that local climatic and agrotechnical conditions can produce field outcomes more than an order of magnitude above the national mean [70].

The practical significance of high-resilience risk classification is further underscored by the scale of control measures involved: nationwide treatment areas against late blight alone ranged from approximately 214,000 to 453,000 hectares annually between 2020 and 2024, reflecting the substantial economic burden associated with high-risk pathogens and pointing to economic-loss modeling as a natural extension of the present methodology [70,71,72,73,74].

Annual monitoring reports consistently attribute year-to-year fluctuations in pathogen spread to prevailing weather conditions, with warm, humid periods repeatedly associated with accelerated disease development [70,71,72,73,74]. This observed sensitivity to climatic variables lends empirical support to climate-scenario modeling of pathogen risk as a direction for future research.

### 4.4. Quarantine Status of High-Risk Pathogens

According to Rosselkhoznadzor’s national quarantine phytosanitary status report, as of the end of 2023 established quarantine zones existed in Russia for the golden potato cyst nematode across 50 federal subjects and 340 municipal districts, covering 509.3 thousand hectares, and for potato wart disease, covering 639 hectares across four regions [75]. Notably, other pathogens assigned comparably high or higher resilience scores in the present classification, including *Ralstonia solanacearum* and *Clavibacter michiganensis* subsp. *sepedonicus*, had no established quarantine zones in Russia as of this reporting period, indicating their current absence or limited detection within national territory despite an inherently high biological risk profile based on resilience parameters alone.

## 5. Conclusions

Based on the analysis and assessment of the level of biological risk according to resilience parameters in the external environment, the following conclusions can be drawn:

1. Low level of risk based on resilience parameters in the external environment is characteristic of 10.9% of the considered pathogens of infectious and parasitic diseases of potato, a medium level of risk is characteristic of 34.8%, a high level of risk of 47.8%, and a very high level of risk of 6.5% of the considered pathogens of IPPD.

2. In the group with a low level of risk based on resilience parameters in the external environment, all pathogens of infectious and parasitic potato diseases have a bacterial etiology. Pathogens of viral, helminth, and mycotic etiology are absent in this group. The average score for the low-risk group is 4.

In the group with a medium level of risk based on resilience parameters in the external environment, the majority of pathogens of IPPD (68.75%) have a viral etiology, 18.75% have a bacterial etiology, and 12.50% have a helminth etiology. Pathogens of mycotic etiology are absent in this group. The average score for the medium-risk group is 5.63. It should be noted that this group included all analyzed representatives of viral etiology, which is due to the peculiarities of the vital activity of viruses and viral complexes.

In the group with a high level of risk based on resilience parameters in the external environment, the majority of pathogens of IPPD (86.36%) have a mycotic etiology, pathogens of bacterial etiology make up 9.09% and pathogens of helminth etiology make up 4.55%. Viral pathogens are absent in this group. The average score for the high-risk group is 8.36. This etiological distribution is associated with the high resilience of mycotic pathogens in the external environment.

In the group with a very high level of risk based on resilience parameters in the external environment, all pathogens of IPPD have a bacterial etiology. The average score for the very high-risk group is 11.67. This group is represented by particularly dangerous pathogens that are free-living inhabitants of the soil.

The analysis of the proportional etiological structure of pathogens of IPPD showed that mycotic pathogens pose the greatest danger in terms of resilience in the external environment. They account for 41.30% of all analyzed pathogens and form the largest share of the high-risk group.

3. The greatest danger to planting material in terms of resilience in the external environment is posed by typical soil-dwelling bacterial pathogens, as well as mycotic and viral pathogens of IPPD.

Typical soil-dwelling bacterial pathogens form the very high-risk group and are particularly dangerous due to their extreme resilience in the external environment, associated with spore and capsule formation.

4. Soil and environmental objects are more significant for the persistence of IPPD pathogens than water systems. The most dangerous in terms of infection of healthy plants are free-living soil inhabitants and pathogens capable of long-term survival in soil in the form of spores, eggs, cysts, and other dormant forms (including viral ones).

5. The analysis of the data shows that among the pathogens of infectious and parasitic potato diseases registered in the Russian Federation over the past five years, the total proportion of infected seed tubers (PIT) was 6.647%, of which mycotic pathogens accounted for 6.381% and helminth pathogens for 0.266%.

The leading place in terms of the level of damage caused to seed potatoes is occupied by pathogens of *Fusarium* (dry rot of tubers)—fungi of the genus *Fusarium*: *F. sambucinum*, *F. solani*, *F. avenaceum*, and *F. oxysporum* (PIT = 1.104%, *Lenv* = 10).

Practical integration of the methodology for identifying the biological risk of the emergence and spread of infectious and parasitic potato diseases involves a systematic assessment of pathogens not only by parameters of resistance in the external environment, which is the subject of this work, but also an assessment of pathogens by parameters of the nature of the course of the disease depending on the bioecological conditions of potato cultivation, which is the subject of further scientific research by the team of authors.

Taken together, these findings confirm the hypothesis formulated in the Introduction: the composite resilience score, derived systematically from literature-reported survival and virulence-retention parameters, correlates significantly with independently recorded field infection data, thereby providing a verifiable, rather than purely expert-based, basis for phytosanitary risk classification.

## Figures and Tables

**Table 1:** Brief characterization of biological risk factors in terms of resilience in the external environment.

№	Risk Factor	Resilience in Water	Resilience in Soil	Resilience in Other Environmental Objects	Resources
1	*Clavibacter michiganensis* subsp. *Sepedonicus* (*Cms*) Quarantine facility in some countries	Can persist in freshwater (contamination).	Lose viability in moist soil after 30–40 days.	In plant residues located in frozen soil, as well as on storage floor surfaces, bags, boxes, and other objects containing residues of air-dry soil, they persist for up to 8 months.	[67,68,69,70,71]
2	Representatives of the genus *Pectobacterium* (*P. carotovorum* subsp. *carotovorum*, *P. atrosepticum*, *P. carotovorum* subsp. *brasiliensis*, *P. wasabiae*)	Can persist in freshwater (contamination).	Can persist in soil (contamination).	Can persist on agricultural equipment (contamination).	[1,2,5,60]
3	Representatives of the genus *Dickeya* (*D. solani*, *D. dianthicola*)	Can persist in freshwater (contamination).	Can persist in soil (contamination).	Characterized by low pathogenicity for three insect species (*Drosophila melanogaster*, *Sitophilus oryzae*, and *Spodoptera littoralis*) and can persist for some time in their dead or living bodies. Can persist in weed residues.	[3,4,5,6,7,8,9,10]
4	*Clostridium perfringens*	More than 1 year (spores).	Free-living soil inhabitants. Spores persist for more than 10 years. Primary habitat.	Persist in manure and feces for more than 1 year.	[1,2,36]
5	*Bacillus cereus*	More than 1 year (spores).	Free-living soil inhabitants. Spores persist for up to 30 years. Primary habitat.	Without disinfection, spores persist on environmental objects (tools, household surfaces, clothing, footwear, etc.) for an indefinite period.	[1,2]
6	*Pseudomonas aeruginosa*	Free-living inhabitants of water.	Free-living soil inhabitants.	In the presence of sufficient nutrients, they remain viable under anaerobic conditions for up to 2 weeks. On cotton and synthetic fabrics, they persist up to 13 days; on semi-woolen fabrics up to 62 days; on felt-like materials more than 150 days; on moist surfaces more than 100 days.	[1,2,34,35]
7	*Ralstonia solanacearium* (*RS*, low-temperature strain *R2b3* is distributed on the territory of the Russian Federation) Quarantine facility	Can persist in freshwater (contamination).	Can persist in soil for up to 1 year; in the presence of large amounts of contaminated plant residues or latently infected tubers, they can persist for more than 1 year.	Can persist in plant residues (up to 3 years), on agricultural equipment (contamination), and on various materials used for potato containers: under cold storage (4 °C), survival on oak wood is 4 days, on poplar 17 days, on high-density polyethylene 48 h, on jute fabric 78 days. In poultry and cattle manure, they persist 2–4 weeks; in potato processing waste 1–2 months. Transmission via clothing is possible.	[67,68,69,70,71,72]
8	*Candidatus Liberibacter solanacearium* (*Lso*) Quarantine facility for EU countries	Are not inhabitants of water systems.	Are not typical inhabitants of soil.	Can persist in living plant tissues (tubers, root crops, bulbs, roots, rhizomes).	[61]
9	Phytoplasmas of the class *Mollicutes* (phytoplasmas of the families *Acholeplasmataceae*: *Potato phytoplasma*, *Mycoplasmataceae* (*Solanaceae phytoplasma PhLO and aster jaundice phytoplasma 16SrI*)	Are not inhabitants of water systems.	Are not typical inhabitants of soil.	Can persist in living plant tissues.	[1,2]
10	*Potato witches’ broom phytoplasma* (class *Mollicutes*, family *Acholeplasmataceae*)	Are not inhabitants of water systems.	Are not typical inhabitants of soil.	Can persist in living plant tissues.	[1,2]
11	*Potato round-leaf phytoplasma* (class *Mollicutes*, family *Acholeplasmataceae*)	Are not inhabitants of water systems.	Are not typical inhabitants of soil.	Can persist in living plant tissues.	[1,2]
12	*Phytoplasmas causing purple curling of the apex*	Are not inhabitants of water systems.	Are not typical inhabitants of soil.	Can persist in living plant tissues.	[1,2]
13	*Globodera rostochiensis* (golden nematode), *Globodera pallida* (pale nematode)	Cysts can be transported by water flows.	In soil, larvae within cysts remain viable for up to 8–9 years. In the absence of a host plant, only a small proportion of larvae emerge from the cysts and subsequently die, while the rest remain in a state of anabiosis until favorable conditions arise.	Can persist in tubers, plant residues, and on agricultural equipment (contamination via infected soil).	[40,41,42]
14	*Ditylenchus destructor Thorne* (stem nematode) Quarantine facility in some countries	Are not inhabitants of water systems.	Can persist for several years in a state of anabiosis; eggs overwinter in soil.	Eggs, larvae, and adults can persist in tubers during storage; eggs may persist in overwintering plants. Infection of healthy tubers may occur via containers, equipment, and sorting facilities, especially in the presence of mechanical damage.	[38,39]
15	*Meloidogyne hapla Chitwood*	Can spread when carried by water flows.	Eggs and larvae can overwinter in soil.	Eggs and larvae can overwinter in neoplasms.	[43]
16	*Potato leafroll virus* (*PLRV*), family *Luteoviridae*, genus *Polerovirus*	Are not typical inhabitants of water systems.	Are not typical inhabitants of soil.	Can persist in living plant tissues, including planting material.	[1,2]
17	*Potato streak virus* (strains: *Yo*, *Yn*, *Yc*, *Yntn*, *Ynw*, *Yz*, *Yze*)	Are not typical inhabitants of water systems.	Are not typical inhabitants of soil.	Can persist in living plant tissues, including planting material.	[57]
18	*Potato virus X*/*Potato latent virus*/*Potato latent leaf spot virus*	Are not typical inhabitants of water systems.	Are not typical inhabitants of soil.	Can persist in plant tissues and planting material.	[53]
19	*Potato virus S* (*SX virus complex*)	Are not typical inhabitants of water systems.	Are not typical inhabitants of soil.	Can persist in plant tissues and planting material.	[1,2]
20	*Potato virus A* (*AX virus complex*)	Are not typical inhabitants of water systems.	Are not typical inhabitants of soil.	Can persist in plant tissues and planting material.	[1,2]
21	*Potato virus* M, *Potato K virus*/*Leaf-rolling mosaic virus* (viruses complexes: *MX*, *KX*, *MS*, *KS*)	Are not typical inhabitants of water systems.	Are not typical inhabitants of soil.	Can persist in plant tissues and planting material.	[1,2]
22	*Potato virus F*/*Potato aucuba mosaic virus*	Are not typical inhabitants of water systems.	Are not typical inhabitants of soil.	Can persist in plant tissues and planting material.	[1,2]
23	*Potato-pseudoaucuba*/*Alfalfa mosaic virus* (*AMV*)	Are not typical inhabitants of water systems.	Are not typical inhabitants of soil.	Can persist in plant tissues and planting material.	[1,2]
24	*Potato virus R*/*Rattle virus*/*Tobacco rattle virus*/*Tobacco mottle virus*/*Internal tuber necrosis virus*	Are not typical inhabitants of water systems.	Are not typical inhabitants of soil.	Can persist in plant tissues and planting material.	[1,2]
25	*Potato mop-top virus* (*PMTV*)	Are not typical inhabitants of water systems.	Spores of *Spongospora subterranea* carrying PMTV can persist in soil for up to 18 years.	Spores can persist in plant tissues and planting material.	[44]
26	*Potato tuber windiness viroid* (*PTWV*)	Are not typical inhabitants of water systems.	Are not typical inhabitants of soil.	Can persist in plant tissues and planting material.	[1,2]
27	Fungi of the genus *Alternaria* (*A. solani*, *A. alternata*, *A. infectoria*, *A. tenuissima*)	Spores are transported by water.	Can persist in plant residues in soil at a depth of no more than 5 cm during the winter period.	Spores can overwinter in plant residues.	[17,18]
28	*Colletotrichum coccodes*	Spores can be transported by water.	Overwinter in soil in the form of sclerotia.	Overwinter in infected tubers and plant residues in the form of sclerotia.	[1,2]
29	Fungi of the genus *Verticillium* (*V. albo-atrum and V. dahliae*)	Spores can be transported by water.	Overwinter in soil in the form of mycelium or microsclerotia.	Overwinter in infected tubers and plant residues in the form of mycelium or microsclerotia.	[17,18]
30	*Polyscytalum pustulans*	Spores can be transported by water.	Spores can persist in soil for 3 to 5 years.	Overwinter in infected tubers and plant residues. Can persist in infected tubers for up to 5 years.	[17,18]
31	*Actinomycetes*: *Streptomyces scabies*	Are not typical inhabitants of water systems.	Typical soil inhabitants (the characteristic smell of freshly plowed soil is due to the presence of streptomycetes producing geosmin).	Streptomycetes causing common scab accumulate mainly in soil on plant residues after harvest.	[17,18]
32	*Myxomycete* (*plasmodiophoromycete*): *Spongospora subterranea*	Amoeboids and plasmodia (several “fused” amoeboids) can move only under conditions of high total moisture, including movement with rainwater flows.	Spores can persist in soil for 3–4 and up to 7 years (at a high level of soil infection).	Overwinter in infected tubers, plant residues, and soil.	[67,68,69,70,71]
33	*Loculoascomycetes*: *Helminthosporium solani*	Total moisture is a growth factor.	Spores can persist in soil with plant residues.	Overwinter in infected tubers. The pathogen mycelium spreads only within the peel cells of the tuber.	[13,14]
34	*Synchytrium endobioticum* Quarantine facility	Total moisture is a growth factor. Infection may be carried from contaminated soil during snowmelt or heavy rainfall.	Spores can persist in soil in the form of zoosporangia for up to 15 years.	Spores overwinter in infected tubers and plant residues.	[15,16]
35	*Basidiomycetes*: *Rhizoctonia solani*	Total moisture is a growth factor.	The fungus can overwinter in soil in the form of mycelium or sclerotia for 3–4 years.	The fungus can overwinter on tubers in the form of mycelium or sclerotia for 3–4 years.	[1,2,17]
36	*Ascomycetes*: *Endomyces geotrichum* (imperfect form of *Geotrichum candidum*)	Total moisture is a growth factor. Reinfection of tubers in storage occurs at temperatures above 10 °C and high humidity.	Spores overwinter in soil.	Spores overwinter in infected tubers, either in storage or in soil.	[17,18]
37	*Oomycetes*: *Phytophthora infestans*	Soil moisture is a factor of growth and transmission of the pathogen. Zoospores can be washed from infected tops into the soil by rain, followed by infection of tubers.	Spores can persist in soil.	Spores overwinter in infected tubers, either in storage or in soil. During winter and also during hot, dry summers, it persists as mycelium in infected tubers, potato stems, and tomato fruits.	[17,18,20,21]
38	*Phoma exigua*	Total moisture is a factor of growth and transmission of the pathogen. In wet weather, pycnidia on stems open, and spores spread by rain and wind, causing new stem infections.	Spores can persist in soil.	Spores overwinter in infected tubers and plant residues.	[22,23]
39	*Fusarium* spp. (*F. sambucinus*, *F. solanaceae*, *F. avenue*, *F. oxysporum*)	Total moisture is a factor of growth and transmission of the pathogen. Sharp fluctuations in temperature and humidity during storage are especially dangerous, leading to tuber sweating.	Typical soil inhabitants. Capable of saprophytic growth on organic residues. Spores overwinter in soil.	Spores overwinter in infected tubers and plant residues. The pathogen persists on agricultural equipment, containers, transport, and in storage facilities if they are not cleaned and disinfected after use.	[24,25]
40	*Oomycetes*: *Pythium ultimum*	Total moisture is a factor of growth and transmission of the pathogen.	The spread of *P. ultimum* spores in soil is facilitated by nematodes. Spores overwinter in soil.	Spores overwinter in infected tubers and plant residues.	[17,18]
41	*Ascochyta lycopersici Brunaud* of the order *Picnidiales* (marsupial stage—*Didymella lycopersici*)	Total moisture is a factor of growth and transmission of the pathogen.	Spores overwinter in soil.	Overwinters in infected tubers and plant residues, persisting as pycnidia and pseudothecia.	[28]
42	*Sclerotinia sclerotiorum*	Total moisture is a factor of growth and transmission of the pathogen.	Spores overwinter in soil. In spring, they germinate in the form of mycelium.	Overwinters in infected tubers and plant residues.	[29]
43	*Erysiphe cichoracearum*	Total moisture is a factor of growth and transmission of the pathogen.	Spores overwinter in soil.	Overwinters in infected plant residues.	[1,2]
44	*Botrytis cinerea* (perfect stage (teleomorpha) of *Botryotinia fuckeliana*)	Total moisture is a factor of growth and transmission of the pathogen. Spores are transported by rain.	Spores overwinter in soil.	Overwinters as sclerotia in infected plant residues.	[31,32]
45	*Passalora concors*	Total moisture is a factor of growth and transmission of the pathogen. Spores are transported by rain.	The fungus can overwinter in soil in the form of thick-walled hyphae and pseudosclerotia.	The fungus persists in plant residues in the form of thick-walled hyphae and pseudosclerotia, which in spring produce new conidial sporulation and serve as a primary source of infection.	[33]
46	*Ustomycete*: *Thecaphora solani* Quarantine object	Are not typical inhabitants of water systems.	Soil is the primary habitat.	The pathogen persists in plant residues and on the surfaces of equipment, agricultural tools, containers, and vehicles.	[17,18]

**Table 2:** Quantitative parameters for assessing the resilience of biological risk factors in the external environment.

Resilience Parameters	Score
Are not typical inhabitants of a given environment (survive for some time, losing virulence and reproductive capacity)	1
Survive for some time (up to a year), retaining virulence (object contamination)	2
Survive for a long time (a year or more), retaining virulence (object contamination)	3
Survive for a long time, retaining virulence and the ability to reproduce in the given environment	4
Are typical inhabitants of a given environment (the environment is favorable for pathogen activity)	5

**Table 3:** Quantitative assessment of the biological risk level based on resilience parameters in the external environment (etiological groups).

№	Risk Factor	Level of Resilience in Water, Lw	Level of Resilience in Soil, Ls	Level of Resilience in Environmental Objects, Lobj	Level of Pathogen Resilience Risk in the External Environment, Lenv	
					Points	Group
1	*Clavibacter michiganensis* subsp. *Sepedonicus* (*Cms*)	2	2	3	7	MR
2	Representatives of the genus *Pectobacterium* (*P. carotovorum* subsp. *carotovorum*, *P. atrosepticum*, *P. carotovorum* subsp. *brasiliensis*, *P. wasabiae*)	2	2	2	6	MR
3	Representatives of the genus *Dickeya* (*D. solani*, *D. dianthicola*)	2	2	2	6	MR
4	*Clostridium perfringens*	3	5	4	12	VHR
5	*Bacillus cereus*	3	5	3	11	VHR
6	*Pseudomonas aeruginosa*	5	5	2	12	VHR
7	*Ralstonia solanacearium* (*RS*, low-temperature strain *R2b3* is distributed on the territory of the Russian Federation)	2	4	3	9	HR
8	*Candidatus Liberibacter solanacearium* (*Lso*)	1	1	2	4	LR
9	Phytoplasmas of the class *Mollicutes* (phytoplasmas of the families *Acholeplasmataceae*: *Potato phytoplasma*, *Mycoplasmataceae*: *Solanaceae phytoplasma PhLO and aster jaundice phytoplasma 16SrI*)	1	1	2	4	LR
10	*Potato witches’ broom phytoplasma* (class *Mollicutes*, family *Acholeplasmataceae*)	1	1	2	4	LR
11	Phytoplasmas of the class *Mollicutes* (phytoplasmas of the families *Acholeplasmataceae*)	1	1	2	4	LR
12	*Phytoplasmas causing purple curling of the apex*	1	1	2	4	LR
13	*Globodera rostochiensis* (golden nematode), *Globodera pallida* (pale nematode)	2	3	3	8	HR
14	*Ditylenchus destructor Thorne* (stem nematode)	1	3	3	7	MR
15	*Meloidogyne hapla Chitwood*	2	2	3	7	MR
16	*Potato virus* L/*Potato leafroll virus* (*PLRV*), family *Luteoviridae*, genus *Polerovirus*	1	1	3	5	MR
17	*Potato virus* Y/*Potato streak virus* (strains: *Yo*, *Yn*, *Yc*, *Yntn*, *Ynw*, *Yz*, *Yze*)	1	1	3	5	MR
18	*Potato X virus*/*Potato latent virus*/*Potato latent leaf spot virus*	1	1	3	5	MR
19	*Potato S virus* (*SX virus complex*)	1	1	3	5	MR
20	*Potato A virus* (*AX virus complex*)	1	1	3	5	MR
21	*Potato M virus*, *Potato K virus*/*Leaf-rolling mosaic virus* (viruses complexes: *MX*, *KX*, *MS*, *KS*)	1	1	3	5	MR
22	*Potato virus F*/*Potato aucuba mosaic virus*	1	1	3	5	MR
23	*Potato-pseudoaucuba*/*Alfalfa mosaic virus* (*AMV*)	1	1	3	5	MR
24	*Potato virus R*/*Rattle virus*/*Tobacco rattle virus*/*Tobacco mottle virus*/*Internal tuber necrosis virus*	1	1	3	5	MR
25	*Potato mop-top virus* (*PMTV*)	1	3	3	7	MR
26	*Potato tuber windiness viroid* (*PSTVd*)	1	1	3	5	MR
27	Fungi of the genus *Alternaria* (*A. solani*, *A. alternata*, *A. infectoria*, *A. tenuissima*)	2	3	3	8	HR
28	*Colletotrichum coccodes*	2	3	3	8	HR
29	Fungi of the genus *Verticillium* (*V. albo-atrum and V. dahliae*)	2	3	3	8	HR
30	*Polyscytalum pustulans*	2	3	3	8	HR
31	*Actinomycetes*: *Streptomyces scabies*	1	5	4	10	HR
32	*Myxomycete* (*plasmodiophoromycete*): *Spongospora subterranea*	2	3	3	8	HR
33	*Loculoascomycetes*: *Helminthosporium solani*	2	3	3	8	HR
34	*Synchytrium endobioticum*	2	3	3	8	HR
35	*Basidiomycetes*: *Rhizoctonia solani*	2	3	3	8	HR
36	*Ascomycetes*: *Endomyces geotrichum* (imperfect form of *Geotrichum candidum*)	2	3	3	8	HR
37	*Oomycetes*: *Phytophthora infestans*	2	3	3	8	HR
38	*Phoma exigua*	2	3	3	8	HR
39	*Fusarium* spp. (*F. sambucinus*, *F. solanaceae*, *F. avenue*, *F. oxysporum*)	2	5	3	10	HR
40	*Oomycetes*: *Pythium ultimum*	2	3	3	8	HR
41	*Ascochyta lycopersici Brunaud* of the order *Picnidiales* (marsupial stage—*Didymella lycopersici*)	2	3	3	8	HR
42	*Sclerotinia sclerotiorum*	2	4	3	9	HR
43	*Erysiphe cichoracearum*	2	3	3	8	HR
44	*Botrytis cinerea* (perfect stage (teleomorpha) of *Botryotinia fuckeliana*)	2	3	3	8	HR
45	*Passalora concors*	2	3	4	9	HR
46	*Ustomycete*: *Thecaphora solani*	1	5	3	9	HR

**Table 4:** Quantitative assessment of the biological risk level based on resilience parameters in the external environment (Risk groups).

№	Risk Factor	Level of Pathogen Resilience Risk in the External Environment, Lenv	
		Points	Group
1	*Candidatus Liberibacter solanacearium* (*Lso*)	4	LR
2	*Pillar wilt phytoplasmas*	4	LR
3	*Potato witches’ broom phytoplasma*	4	LR
4	*Potato round-leaf phytoplasma*	4	LR
5	*Phytoplasmas causing purple curling of the apex*	4	LR
6	*Potato X virus*/*Potato latent leaf spot virus*	5	MR
7	*Potato S virus* (*SX virus complex*)	5	MR
8	*Potato A virus* (*AX virus complex*)	5	MR
9	*Potato M virus*, *Potato K virus*/*Leaf-rolling mosaic virus* (viruses complexes: *MX*, *KX*, *MS*, *KS*)	5	MR
10	*Potato virus F*/*Potato aucuba mosaic virus*	5	MR
11	*Potato-pseudoaucuba*/*Alfalfa mosaic virus* (*AMV*)	5	MR
12	*Potato virus R*/*Rattle virus*	5	MR
13	*Potato tuber windiness viroid* (*PSTVd*)	5	MR
14	*Potato virus* L/*Potato leafroll virus* (*PLRV*)	5	MR
15	*Potato virus* Y/*Potato streak virus* (strains: *Yo*, *Yn*, *Yc*, *Yntn*, *Ynw*, *Yz*, *Yze*)	5	MR
16	*Pectobacterium* spp.	6	MR
17	*Dickeya* spp.	6	MR
18	*Clavibacter michiganensis* subsp. *Sepedonicus* (*Cms*)	7	MR
19	*Ditylenchus destructor Thorne*	7	MR
20	*Meloidogyne hapla Chitwood*	7	MR
21	*Potato mop-top virus* (*PMTV*)	7	MR
22	*Globodera rostochiensis*, *Globodera pallida*	8	HR
23	*Alternaria* spp.	8	HR
24	*Colletotrichum coccodes*	8	HR
25	*Verticillium* spp.	8	HR
26	*Polyscytalum pustulans*	8	HR
27	*Spongospora subterranea*	8	HR
28	*Helminthosporium solani*	8	HR
29	*Synchytrium endobioticum*	8	HR
30	*Rhizoctonia solani*	8	HR
31	*Endomyces geotrichum*	8	HR
32	*Phytophthora infestans*	8	HR
33	*Phoma exigua*	8	HR
34	*Oomycetes*: *Pythium ultimum*	8	HR
35	*Ascochyta lycopersici Brunaud*	8	HR
36	*Erysiphe cichoracearum*	8	HR
37	*Botrytis cinerea*	8	HR
38	*Ralstonia solanacearium* (*RS*, *strain R2b3*)	9	HR
39	*Sclerotinia sclerotiorum*	9	HR
40	*Passalora concors*	9	HR
41	*Thecaphora solani*	9	HR
42	*Streptomyces scabies*	10	HR
43	*Fusarium* spp.	10	HR
44	*Bacillus cereus*	11	VHR
45	*Pseudomonas aeruginosa*	12	VHR
46	*Clostridium perfringens*	12	VHR

**Table 5:** Relationship between the risk level of infection with infectious and parasitic potato diseases (IPPD) based on transmission mechanisms and pathways (*Ltr*) and resilience in the external environment (*Lenv*).

Risk Factors (Pathogens of Infectious and Parasitic Potato Diseases)	Level of Transmission Risk of the Pathogen (Ltr), Points	Level of Pathogen Resilience Risk in the External Environment (Lenv), Points
*Helminthosporium solani*	10.9	8.0
*Globodera rostochiensis*	10.7	8.0
*Globodera pallida*	10.7	8.0
*Rhizoctonia solani*	10.6	8.0
*Phoma exigua*	10.6	8.0
*Phytophthora infestans*	10.6	8.0
*Colletotrichum coccodes*	10.6	8.0
*Sclerotinia sclerotiorum*	10.6	9.0
*Fusarium* spp.	10.6	10.0

**Table 6:** Dynamics of infection of seed potato tubers by the main pathogens of infectious and parasitic diseases.

Risk Factor	Level of Pathogen Resilience Risk in the External Environment (Lenv)		Proportion of Affected Tubers, %					
	Points	Group	2020	2021	2022	2023	2024	Average Annual Value for the Period 2020–2024
*Helminthosporium solani*	8	HR	0.190	0.180	0.080	0.100	0.040	0.118
*Globodera rostochiensis*	8	HR	0.110	0.120	0.190	0.180	0.030	0.126
*Globodera pallida*	8	HR	0.160	0.150	0.120	0.150	0.120	0.140
*Rhizoctonia solani*	8	HR	0.170	0.350	0.590	0.530	0.500	0.428
*Phoma exigua*	8	HR	0.620	0.630	0.450	0.450	0.610	0.552
*Phytophthora infestans*	8	HR	0.970	1.100	0.880	0.930	1.024	0.981
*Colletotrichum coccodes*	8	HR	1.200	1.120	0.950	1.200	0.520	0.998
*Sclerotinia sclerotiorum*	9	HR	1.120	1.050	1.110	1.130	1.080	1.098
*Passalora concors*	9	HR	1.080	1.120	1.110	1.030	1.170	1.102
*Fusarium* spp.	10	HR	1.070	1.100	1.280	0.930	1.140	1.104

**Table 7:** Validation against field-level spread (average for 2020–2024) for pathogens and groups of pathogens, based on monitoring data from Rosselkhoztsentr over five years.

Risk Factor	Lenv, Points	Mean Field-Level Spread Area, % (2020–2024)	Group
*Phytophthora infestans*	8	6.95	HR
*Alternaria* spp.	8	5.51	HR
*Rhizoctonia solani*	8	2.46	HR
*Pectobacterium* spp.	6	0.23	MR
Viral diseases	5.36	0.40	MR

## Data Availability

The original contributions presented in this study are included in the article. Further inquiries can be directed to the corresponding author.
